# Quantifying patient preferences for inpatient pharmacist consultation: a multi-center discrete choice experiment in Sichuan, China

**DOI:** 10.3389/fpubh.2026.1809396

**Published:** 2026-05-05

**Authors:** Guirong Xiao, Di Xiao, Liyuan Liu, Yanhua Chen, Ming Hu

**Affiliations:** 1 Department of Pharmacy, West China Hospital, Sichuan University, Chengdu, Sichuan, China; 2 Department of Pharmacy, Chengdu Shangjin Nanfu Hospital, Chengdu, Sichuan, China; 3 West China School of Pharmacy, Sichuan University, Chengdu, Sichuan, China; 4 Department of Pharmacy, Hospital of Chengdu University of Traditional Chinese Medicine, Chengdu, Sichuan, China

**Keywords:** clinical pharmacist, discrete choice experiment, inpatient pharmacist consultation, pharmaceutical services, preference, willingness-to-pay

## Abstract

**Background:**

Pharmaceutical services play a critical role in safeguarding medication safety and optimizing therapeutic outcomes. Their value is increasingly recognized in China, where recent policy reforms have introduced new medical service pricing items, and several provinces have piloted an inpatient consultation fee (clinical pharmacy surcharge), namely inpatient pharmacist consultation (IPC), as a billable services.

**Objective:**

This study used a discrete choice experiment (DCE) to evaluate hospitalized patients’ preferences for IPC and the factors shaping these choices, providing evidence to inform service development and policy-making.

**Methods:**

Study attributes and levels were derived from literature review, patient surveys, and expert interviews. Attributes included hospital tier (tertiary vs. non-tertiary), pharmacist professional title (attending pharmacist, associate chief pharmacist, chief pharmacist), consultation frequency (once, three times, five times per week), duration (5, 10, 15 min), service outcome (improved efficacy vs. reduced medication errors), and unit cost (10 or 15 CNY per session; 1 CNY ≈ 0.15 USD). A DCE questionnaire was administered to inpatients across six Sichuan hospitals. Preferences were analyzed using a mixed logit model to estimate regression coefficients (*β*) and willingness-to-pay (WTP).

**Results:**

Of 320 valid responses, patients preferred consultations delivered by associate chief pharmacists (*β* = 0.182, *p* < 0.001; WTP 5.00 CNY) in tertiary hospitals (*β* = 0.138, *p* < 0.001; WTP 3.80 CNY), with a primary focus on reducing medication errors (*β* = 0.125, *p* = 0.002; WTP 3.42 CNY). Patients favored three sessions per week (*β* = 0.156, *p* = 0.005; WTP 4.29 CNY) lasting 15 min each (*β* = 0.133, *p* = 0.016; WTP 3.65 CNY). Service cost had a limited negative influence on choices (*β* = −0.036, *p* < 0.001) within the narrow price range of 10 to 15 CNY tested in this study. The optimal profile (associate chief pharmacist, tertiary hospital, three times per week, 15 min/session, error reduction focus, 10 CNY/session) achieved a 68.4% predicted choice probability. Subgroup analyses showed that low-income patients prioritized consultation frequency, urban patients preferred longer duration, and patients with chronic diseases emphasized efficacy over error reduction.

**Conclusion:**

Inpatients demonstrated clear preferences for attributes of IPC, with variations by income, residence, and chronic disease status. Greater involvement of clinical pharmacists, tailored adjustment of consultation frequency and duration, and integration into hierarchical diagnosis and treatment and chronic disease management were encouraged. Price has limited influence on choices. We recommend including pharmaceutical consultations in health insurance reimbursement after pilot programs.

## Highlights

- Grounded in patients’ authentic needs and value orientations, this study utilised DCE methodology to quantify relative preference weights and WTP for each attribute level. The estimated preference weights provide direct quantitative evidence for designing interventions that concurrently ensure medication safety/efficacy, heighten patient satisfaction, and enhance the efficiency of resource allocation—thereby advancing IPC towards closer alignment with patient-centred care frameworks.
- Stratified analyses revealed significant preference heterogeneity: low-income patients assigned higher priority to consultation frequency, urban patients favored extended consultation duration, and patients with chronic diseases demonstrated a greater relative emphasis on efficacy compared to error reduction. Recognising these nuanced distinctions is crucial for providing individualized services and maximising the contribution of clinical pharmacists.

## 1 Introduction

The evolution of pharmacy services in China is characterised by two fundamental shifts: a transition from drug-centred to patient-centred care, and a move from supply assurance towards professional service delivery and clinical engagement. Clinical pharmacists, aligned with patient needs and therapeutic goals, play a pivotal role in safeguarding medication safety and optimizing treatment outcomes (1, 2). In 2023, the National Healthcare Security Administration introduced national-level pricing items for pharmacy-related services, encompassing outpatient consultation fees, inpatient consultation fees (clinical pharmacy surcharge), and in-hospital consultation fees. The technical and labor value of pharmacy services gained progressive recognition in provincial pilot programs, and 15 provinces issued notices establishing a sub-item under inpatient consultation fees—“inpatient consultation fee (clinical pharmacy surcharge),” namely inpatient pharmacist consultation (IPC).

The IPC entails collaboration between clinical pharmacists and physicians during inpatient consultations. They integrate disease assessment, medication review, and laboratory findings to design individualized treatment plans. Key pharmacist activities include dosage calculation, medication reconciliation, therapeutic drug monitoring, adverse drug reaction surveillance, clinical intervention implementation, and clinical progression documentation within medical records. The IPC emphasises active clinical engagement through participation in ward rounds, prescription review, patient education, and pharmaceutical monitoring to ensure safe and effective therapeutic regimens.

Currently, the IPC are being piloted mainly in some tertiary public hospitals across 15 provincial-level administrative divisions. To explore patient preferences, we surveyed multiple inpatients who had experienced IPC during hospitalization: “What type of IPC do you prefer?” Responses varied by pharmacist professional title, service outcomes, and cost, reflecting the multidimensional trade-offs inherent in patient decision-making. Traditional methods struggle to capture these subjective preferences quantitatively, whereas DCE provide an efficient method.

Rooted in random utility theory and stated-preference methodology, DCE elicits individuals’ preferences by presenting hypothetical scenarios with systematically varied attributes and levels, thereby uncovering the trade-offs and decision patterns guiding their choices. As a method widely applied in healthcare, transportation, and environmental research, DCE has become an established method for assessing preferences of patients, clinicians, and policymakers regarding treatment options, health services, and health policies, generating empirical evidence to optimize these treatments, services, and policies (3, 4). For instance, studies of adults with type 2 diabetes across Canada, Spain, France, and Japan demonstrated that injection frequency, hypoglycemia risk, and timing influenced preferences for basic insulin therapy, with injection frequency being the greatest influence (5). Another study on pharmacogenetic testing in medically underserved populations found that out-of-pocket cost was the most important determinant, whereas wait time was least relevant, underscoring the need to address affordability and insurance coverage (6).

This study employed DCE to quantify patient preferences for IPC, identify key determinants, estimate willingness-to-pay (WTP) for specific attributes, and conduct subgroup analyses, thus translating abstract service features into tangible values and monetary terms. This study was approved by the ethics committee of West China Hospital, Sichuan University (protocol code 2026-185).

## 2 Materials and methods

### 2.1 Theoretical basis of the discrete choice experiment

The methodological basis of DCE originates from the random utility model framework (7). Under this framework, the utility of each alternative for a respondent comprises a systematic component ( V ) and a random component ( ε ). The systematic component reflects stable preferences over observable attributes, which are operationalized as a set of measurable characteristics, such as hospital tier, pharmacist professional title, and service cost. Regression coefficients ( β ) indicate the relative importance of each attribute; larger absolute values denote stronger effects on choice, and the sign of β reflects the direction of influence. ε accounts for stochastic variation and unobserved influences, combining latent factors with measurement error. Thus, the total utility $U_{\mathrm{nc}}$ that respondent n obtained from alternative c is expressed as:


$$U_{\mathrm{nc}}=V_{\mathrm{nc}}+\varepsilon_{\mathrm{nc}}=\beta_{0}+\beta_{1}X_{1\mathrm{nc}}+\beta_{2}X_{2\mathrm{nc}}+\cdots +\beta_{m}X_{\mathrm{mnc}}+\varepsilon_{\mathrm{nc}}$$

In general, respondents are assumed to select the alternative that yields the highest perceived utility. For example, the probability that respondent *n* chose option a over option b is represented as:


$$\begin{array}{l} P_{\mathrm{na}}=P_{r}(U_{\mathrm{na}}>U_{\mathrm{nb}})=P_{r}(V_{\mathrm{na}}+\varepsilon_{\mathrm{na}}>V_{\mathrm{nb}}+\varepsilon_{\mathrm{nb}})\\ =P_{r}(\varepsilon_{\mathrm{na}}-\varepsilon_{\mathrm{nb}}>V_{\mathrm{nb}}-V_{\mathrm{na}}) \end{array}$$

A variety of discrete choice models have been developed for parameter estimation, differing primarily in their distributional assumptions regarding the error term and their ability to account for preference heterogeneity (8). The conditional logit model, formalized by McFadden in 1974, assumes an independently and identically distributed extreme-value error term. Although computationally efficient, it is constrained by the independence of irrelevant alternatives (IIA) assumption, which is often violated in empirical settings. More flexible approaches were therefore introduced to address unobserved heterogeneity. The mixed logit model, or random parameters logit model, allows coefficients to vary across individuals according to a continuous distribution, thereby capturing preference heterogeneity and fully relaxing the IIA restriction (9). This specification effectively captures heterogeneous preferences and enables interactions among attributes, leading to more reliable estimates of choice behavior (10, 11). This study adopted the mixed logit model for fitting and analysis.

Since the choice probability involves an integral without a closed-form solution, it is estimated via simulation methods, typically using Halton draws. Repeated random samples are generated from the assumed distribution of coefficients, and the corresponding logit probabilities are averaged to obtain consistent estimates of individual choice probabilities (12).

### 2.2 Determination of attributes and levels

Discrete choice experiment requires precise definition of attributes and levels. Attributes and levels were determined through policy review, literature analysis, expert interviews, and patient surveys for this study.

First, we reviewed policy documents issued by provincial healthcare security administrations that had initiated pilot programs. Provincial differences were observed. Most provinces restricted implementation to tertiary public hospitals, with the exception of Fujian and Guangxi. Pharmacist professional titles were limited to attending pharmacists or higher. Although this requirement was not consistently specified in provincial documents, national pharmaceutical service guidelines mandated that activities such as medication education be conducted by pharmacists at the attending level or above. Pricing policies generally followed a “per-session fee with capped maximum” approach. Services were charged daily at 8–15 CNY (mainly 10 CNY), with the maximum number of billable sessions determined by hospitalization length. Total fee caps ranged from 100 to 150 CNY. Table 1 presents the charging standards for IPC in pilot provinces.

**Table 1:** Policies and implementation status of IPC service pilots across Chinese provinces.

Provinces	Hospital tier	Pharmacist professional title	Unit cost (CNY)	Maximum number of billable times	
				Length of stay ≤30 days	Length of stay >30 days
Sichuan	Tertiary public	Clinical Pharmacists	15	6	10
Chongqing	Tertiary public	Clinical Pharmacists	10	6	10
Hebei	Tertiary public	Clinical Pharmacists	14	3	7
Jiangxi	Tertiary public	Pharmacists at the Attending Level or Above	10	6	10
Hunan	Tertiary public	Clinical Pharmacists	10	6	10
Fujian	Public (grade 3A and below)	Clinical Pharmacists	10	6	10
Hubei	Tertiary public	Pharmacists at the Attending Level or Above	10	6	10
Shandong	Tertiary	Clinical Pharmacists	14	3	10
Beijing	Tertiary public	Clinical Pharmacists	10	6	10
Guangxi Zhuang Autonomous Region	Public	Clinical Pharmacists	Tertiary Hospital 10/d, Secondary Hospital 9/d, Primary or Below 8/d	Total ≤60 CNY	Total ≤100 CNY
Shaanxi	Tertiary public	Clinical Pharmacists	10	6	10
Hainan	Public	Clinical Pharmacists	Tertiary Hospital 12/d, Secondary Hospital 11/d, Primary or Below 10/d	Total ≤36 CNY	Total ≤120 CNY
Henan	Public (grade 3A and below)	Clinical Pharmacists	10	5	10
Jilin	Ministry- and provincial-level	Clinical Pharmacists	12	3	10
Inner Mongolia Autonomous Region	Inner Mongolia People’s Hospital and The Affiliated Hospital of Inner Mongolia Medical University	Clinical Pharmacists	10	6	10

Secondly, PubMed was searched using terms including “patient preference,” “pharmaceutical care,” and “discrete choice experiment”. Relevant preference studies predominantly focused on community pharmacy, primary health care, specific diseases, or treatment regimens; no studies examining preferences for IPC were identified (13–16). Attributes potentially applicable to IPC were preliminarily extracted extracted, including service provider, service content (professional guidance), service duration, service frequency, continuity of care, service attitude, service outcome (improved efficacy, reduced adverse events), and service cost (13–16).

Finally, integrating policy documents, literature review, and expert interviews, the research team established 6 attributes: including provider-related attributes (hospital level, pharmacist professional title), process attributes (consultation duration, consultation frequency), an outcome attribute (service outcome), and service cost, with 2 to 3 levels set for each attribute. Consistent with relevant policies and the current pilot implementation primarily in some tertiary public hospitals, hospital level was categorized into tertiary public hospitals and non-tertiary public hospitals. Pharmacist professional titles were stratified into three levels: attending pharmacist, associate chief pharmacist, and chief pharmacist. Service cost was set at 10 CNY per visit and 15 CNY per visit (1 CNY ≈ 0.15 USD).

The service outcome attribute comprised two levels reflecting patients’ perceptions of clinical pharmacists’ core roles:

Reduced medication errors: representing the pharmacist as a safety guardian who intercepts errors, prevents adverse events, and safeguards medication safety.

Improved efficacy: representing the pharmacist as a treatment optimizer who adjusts regimens to maximize therapeutic effectiveness.

This dichotomous setting was applied to facilitate clear trade-offs in the DCE. Based on pre-survey results and patient feedback, three levels were were assigned to both consultation frequency and consultation duration. Details are presented in Table 2.

**Table 2:** Attributes and levels for the DCE.

Number	Attributes	Definition	Levels
1	Hospital tier	In China, hospitals are officially classified into a three-tier system based on their function and tasks, as mandated by the Hospital Grading Management Approach issued by the Ministry of Health. Tertiary hospitals represent the highest standard of medical service and comprehensive capacity in China. Non-tertiary public hospitals consist of secondary hospitals (e.g., municipal and county-level general hospitals) and primary hospitals (e.g., community health centers and township health centers).	Tertiary public hospitals
			Non-tertiary public hospitals
2	Pharmacist professional title	Pharmacist professional titles in China comprise five ranks, but only attending pharmacists and above met eligibility requirements for clinical service. To aid patient comprehension, explanatory notes on professional competence were provided.	Chief Pharmacist (“Senior Specialist”)
			Associate Chief Pharmacist (“Specialist”)
			Attending Pharmacist (“General”)
3	Service outcome	Service outcome reflect patients’ expectations following consultation.	Reduced medication errors
			Improved efficacy
4	Consultation duration	Duration captures the time pharmacists spent on ward-based activities such as medication evaluation, prescription review, and patient education, with notes clarifying the depth of communication.	5 min/session (Brief)
			10 min/session (Standard)
			15 min/session (Comprehensive)
5	Consultation frequency	Frequency denotes the number of consultations within a defined period.	once per week
			Three times per week
			Five times per week
6	Unit cost	Unit cost represents the professional fee charged per session.	10 CNY
			15 CNY

### 2.3 Questionnaire development

**Figure 1:**
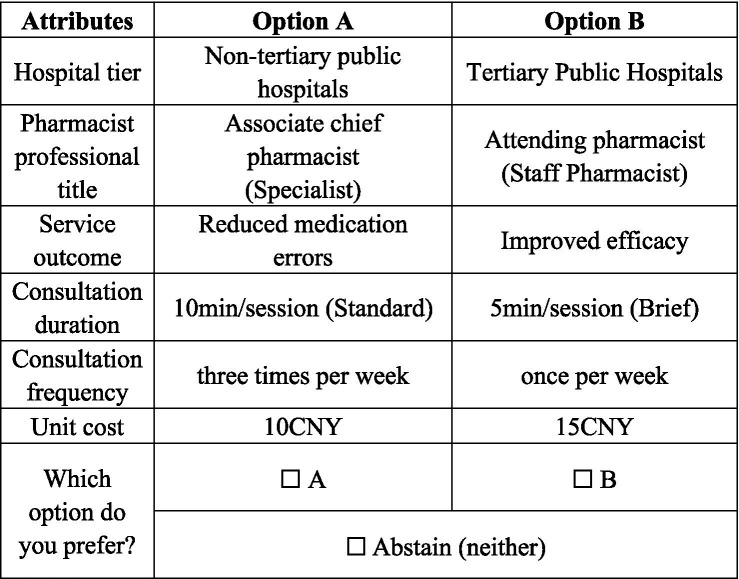
Example of a choice set.

Following the determination of attributes and levels, the questionnaire was designed. Six attributes were included: three with two levels (unit cost, service outcome, hospital tier) and three with three levels (pharmacist professional title, consultation frequency, consultation duration). Orthogonal design via Ngene generated 36 choice sets, distributed across four questionnaire versions (9 scenarios + 1 consistency check). The first scenario in each version was duplicated as the tenth to evaluate internal consistency (17). A sample scenario is illustrated in Figure 1.

The questionnaire comprised two sections. The first captured demographics and clinical context: sex, age, education, residence, inpatient department, chronic conditions requiring long-term therapy, number of medications, insurance type, household per capita disposable income, and prior exposure to IPC. The second comprised the DCE tasks.

### 2.4 Sample size determination

We used the Johnson–Orme rule (18) to determine the minimum sample size required for the DCE. The equation is:


N≥500c/(t×a)

where:

*c* = number of levels per attribute (3),
*t* = number of choice tasks per respondent (9),
*a* = number of alternatives per task (2).

The minimum calculated sample size was 84. To enable subgroup analyses and ensure precision, we aimed to recruit at least 300 valid responses.

### 2.5 Survey administration and quality control

At present, IPC is piloted in a limited number of hospitals in China, so strict random sampling was not feasible. To maximize sample representativeness, hospitals were purposively selected to encompass different regions (urban, prefecture-level, and minority areas) and hospital types (general hospitals and traditional Chinese medicine hospitals). The DCE questionnaire was administered to inpatients across six hospitals in Sichuan province: West China Hospital, Sichuan University; Ziyang Central Hospital; Chengdu Shangjin Nanfu Hospital; Hospital of Chengdu University of Traditional Chinese Medicine (Traditional Chinese Medicine Hospital of Sichuan Province); the First Hospital of Liangshan; and West China Tianfu Hospital, Sichuan University.

All investigators received standardized training before data collection. Pilot testing revealed that respondents struggled to comprehend pharmacist titles; thus, for clarity, the titles “attending pharmacist,” “associate chief pharmacist,” and “chief pharmacist” were annotated as “general pharmacist,” “specialist,” and “senior specialist,” respectively. During the formal survey, participants provided informed consent and completed a QR-code–based questionnaire. Investigators assisted respondents as needed and instructed them to choose their preferred profile from three alternatives: Option A, Option B, or abstain (choose neither). Participants could withdraw at any point; those who proceeded submitted the questionnaire upon completion.

### 2.6 Data statistics and analysis method

We collected questionnaires, formatted the raw data, and analyzed responses using a mixed logit model in R version 4.3.

Analysis focused on two dimensions. Coefficients were interpreted against base-level attributes, with each β reflecting the average utility change associated with selecting a non-reference level (19, 20). By introducing the cost variable, WTP was estimated to express preference differences in monetary terms (21, 22). WTP was calculated as:


$$\mathrm{WTP}(X)=-\frac{\partial U/\partial X}{\partial U/\partial \text{cost}}=-\frac{\beta_{X}}{\beta_{M}}$$

Where $\beta_{X}$ is the coefficient associated with attribute X , and $\beta_{M}$ is the coefficient for the cost variable.

Attribute relative importance was determined by calculating the discrepancy between its most favored and least favored levels, then multiplying this range by the attribute’s respective regression coefficient. These values were subsequently normalized to support cross-attribute comparison. A relative importance score represents the relative contribution of an attribute when shifting from its least desirable to most desirable level. Higher scores signify stronger influence on preference formation (23).

Based on the parameter estimates of the mixed logit model constructed in R software, predicted choice probabilities were computed using the standard logit formula. Uptake rate—defined as the difference in predicted choice probability between a modified scenario (one attribute level altered) and the baseline scenario—quantified changes per attribute level. The optimal profile was constructed by sequentially introducing each attribute’s optimal level, and its predicted choice probability was then calculated (24).

## 3 Results

### 3.1 Demographic characteristics

A total of 355 questionnaires were distributed and returned, yielding a 100% response rate. After excluding 35 invalid questionnaires due to inconsistent responses between scenarios 1 and 10 or completion time under 300 s, 320 valid questionnaires were analyzed, representing an effective rate of 90.14%.

Table 3 summarizes respondent characteristics. Women comprised 65.3% of the sample, and most participants were from non-surgical departments (73.8%). The majority were young adults aged 18–44 years (75.0%). Chronic conditions requiring long-term therapy were reported by 31.2% of respondents, while polypharmacy (≥5 medications) was observed in 12.8%. Most respondents had attained a bachelor’s degree or higher (79.7%), lived in urban areas (78.1%), and were insured primarily through the urban employee scheme (60.6%). Annual household per capita disposable income was ≥40,000 CNY for 75.0% of respondents.

**Table 3:** Respondents’ demographic characteristics (*n* = 320).

Characteristic	No.	Percentage (%)
Sex		
Men	111	34.7
Women	209	65.3
Age		
18–44	240	75
45–64	59	18.4
≥65	21	6.6
Education		
Below bachelor’s degree	65	20.3
Bachelor’s degree or higher	255	79.7
Residence		
Urban	250	78.1
Rural	70	21.9
Inpatient department		
Surgical	84	26.2
Non-surgical	236	73.8
Chronic conditions requiring long-term therapy		
Yes	100	31.2
No	220	68.8
Number of medications		
≤4	279	87.2
≥5	41	12.8
Insurance type		
Urban employee basic medical insurance	194	60.6
Urban and rural resident basic medical insurance	126	39.4
Household per capita disposable income		
<40,000/year	80	25
≥40,000/year	240	75

### 3.2 Patient preferences and attribute importance

**Figure 2:**
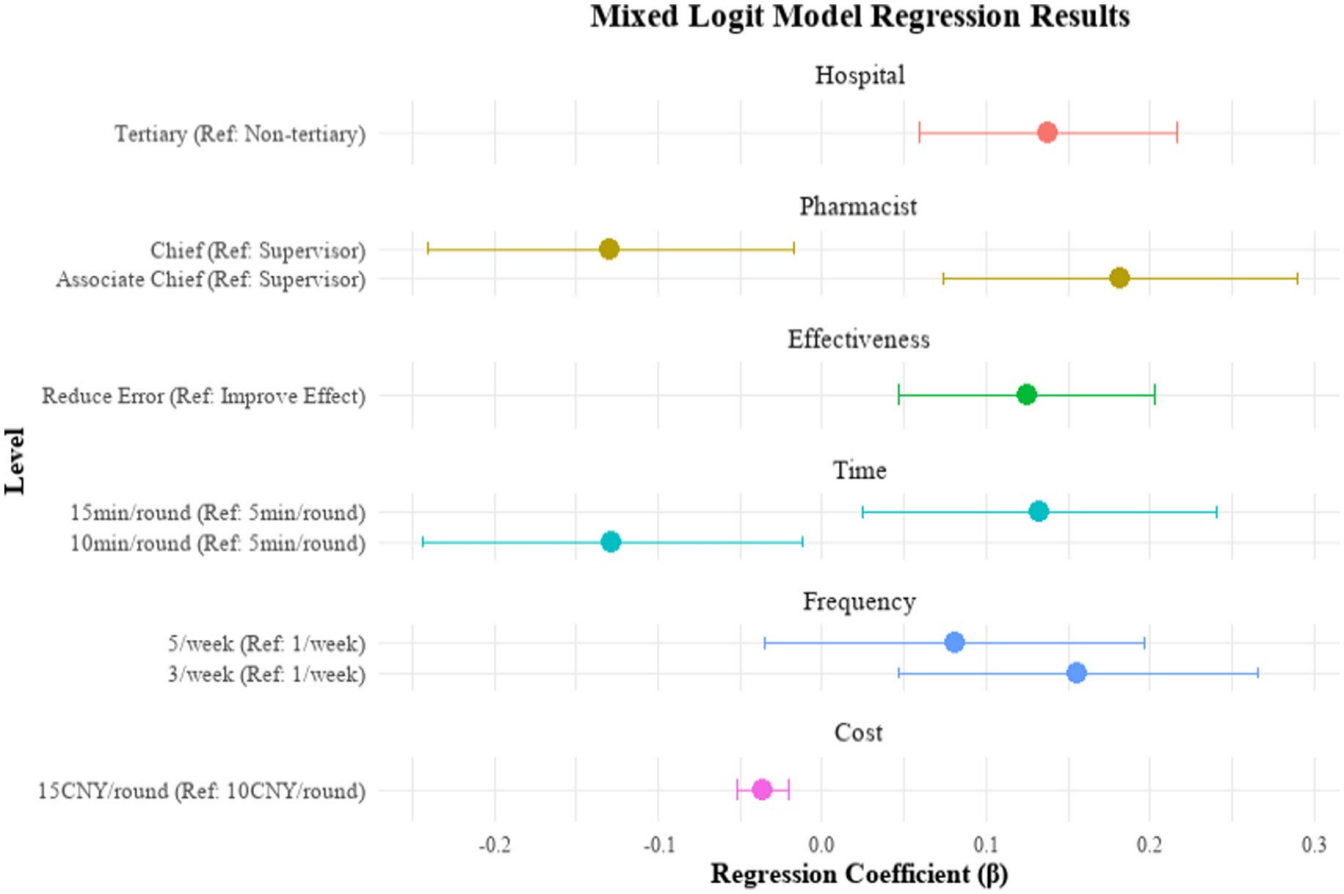
Forest plot of the mixed logit model regression results.

Mixed logit estimates are shown in Table 4 and Figure 2. Patients expressed a clear preference for tertiary hospitals (*β* = 0.138, *p* < 0.001) and for services delivered by associate chief pharmacists (*β* = 0.182, *p* < 0.001). The outcome “reducing medication errors” was positively valued (*β* = 0.125, *p* = 0.002). Cost negatively influenced choice, with a fee of 15 CNY per session significantly less preferred than 10 CNY (*β* = −0.036, *p* < 0.001), reflecting patient sensitivity to price. Longer consultations of 15 min (*β* = 0.133, *p* = 0.016) and a frequency of three sessions per week (*β* = 0.156, *p* = 0.005) were also associated with greater preference.

**Table 4:** Mixed logit model of preferences for IPC among patients.

Attributes and levels	*β* (SE)	*p*
Hospital tier (Ref: non-tertiary public hospitals)		
Tertiary public hospitals	0.138 (0.040)	<0.001
Pharmacist professional title (Ref: Attending Pharmacist)		
Associate Chief Pharmacist	0.182 (0.055)	<0.001
Chief Pharmacist	−0.129 (0.057)	0.024
Consultation frequency (Ref: once per week)		
Three times per week	0.156 (0.056)	0.005
Five times per week	0.081 (0.059)	0.167
Consultation duration (Ref: 5 min/session)		
10 min/session	−0.128 (0.059)	0.029
15 min/session	0.133 (0.055)	0.016
Service outcome (Ref: improved efficacy)		
Reduced medication errors	0.125 (0.040)	0.002
Unit cost (Ref: 10CNY)		
15CNY	−0.036 (0.008)	<0.001
Sample size	320	
Log likelihood	−1,854.7	
Likelihood ratio test	*χ*^2^ = 146.75	<0.001
Akaike information criterion (AIC)	3,735.4	
Bayesian information criterion (BIC)	3,881	

*β*, the average preferences of the study population; SE, standard error; Ref, reference level.

**Figure 3:**
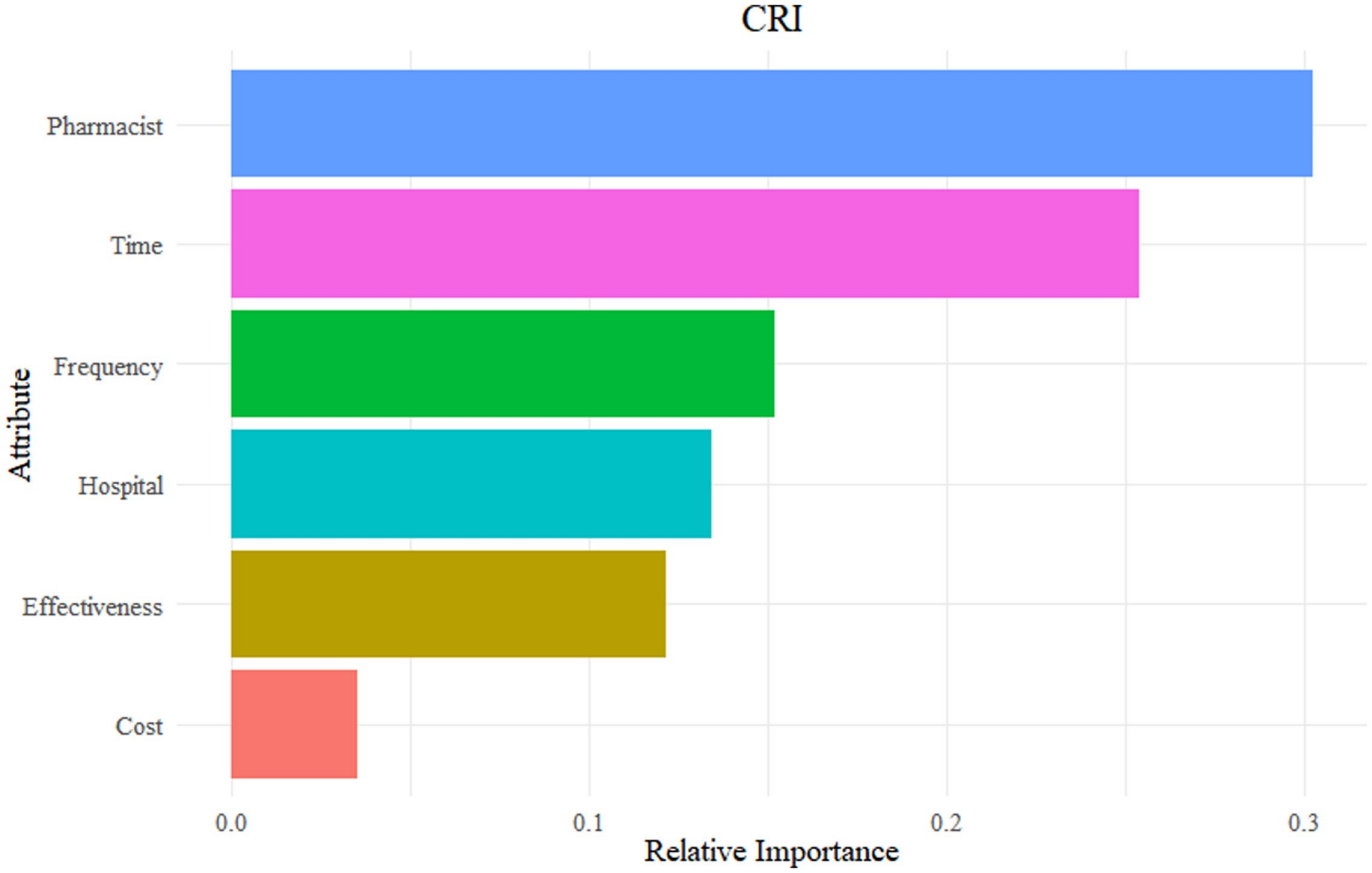
Relative importance scores of attributes on respondents’ decision-making.

Figure 3 illustrates marked differences in attribute importance. Pharmacist professional title was the most influential attribute (relative importance: 30.3%), followed by duration (25.4%) and frequency (15.2%). Hospital tier (13.4%) and service outcome (12.2%) were of intermediate importance, whereas service cost contributed minimally (3.5%).

### 3.3 Willingness-to-pay

Table 5 reports WTP estimates derived from mixed logit analysis. Patients were willing to pay an additional 5.00 CNY (95% CI, 2.56–7.44) for services provided by associate chief rather than attending pharmacists, indicating the decisive role of professional qualification. Preference for tertiary hospitals translated into an incremental WTP of 3.80 CNY (95% CI, 1.83–5.77). Increasing consultation frequency from once to three times per week raised WTP by 4.29 CNY (95% CI, 1.82–6.76), while extending duration from 5 to 15 min increased WTP by 3.65 CNY (95% CI, 0.91–6.39). For service outcomes, reducing medication errors was valued 3.42 CNY higher per session (95% CI, 1.45–5.39) than improving efficacy, underscoring patients’ prioritization of medication safety.

**Table 5:** Respondents’ WTP for IPC services.

Attributes and levels	*p*	WTP/CNY	95% Cl	Attributes and levels
Hospital tier (Ref: non-tertiary public hospitals)				
Tertiary public hospitals	<0.001	3.8	1.83	5.77
Pharmacist professional title (Ref: Attending Pharmacist)				
Associate Chief Pharmacist	<0.001	5	2.56	7.44
Chief Pharmacist	0.024	−3.54	−6.69	−0.39
Consultation frequency (Ref: once per week)				
Three times per week	0.005	4.29	1.82	6.76
Five times per week	0.167	2.23	−0.93	5.39
Consultation duration (Ref: 5 min/session)				
10 min/session	0.029	−3.5	−6.57	−0.43
15 min/session	0.016	3.65	0.91	6.39
Service outcome (Ref: improved efficacy)				
Reduced medication errors	0.002	3.42	1.45	5.39

CI, confidence interval.

### 3.4 Predicted choice probabilities

The uptake rate was quantified as the change in predicted choice probability relative to the baseline scenario (non-tertiary hospital, attending pharmacist, improved efficacy, 5 min/session, once per week, 10 CNY per session). Compared with the baseline, the largest increase in choice probability was observed for consultations delivered by associate chief pharmacists (+4.54%), followed by three consultations per week (+3.89%), tertiary hospitals (+3.44%), 15 min/session (+3.32%), and reducing medication errors (+3.12%). A fee increase from 10 to 15 CNY per session produced a modest decline (−0.90%).

**Figure 4:**
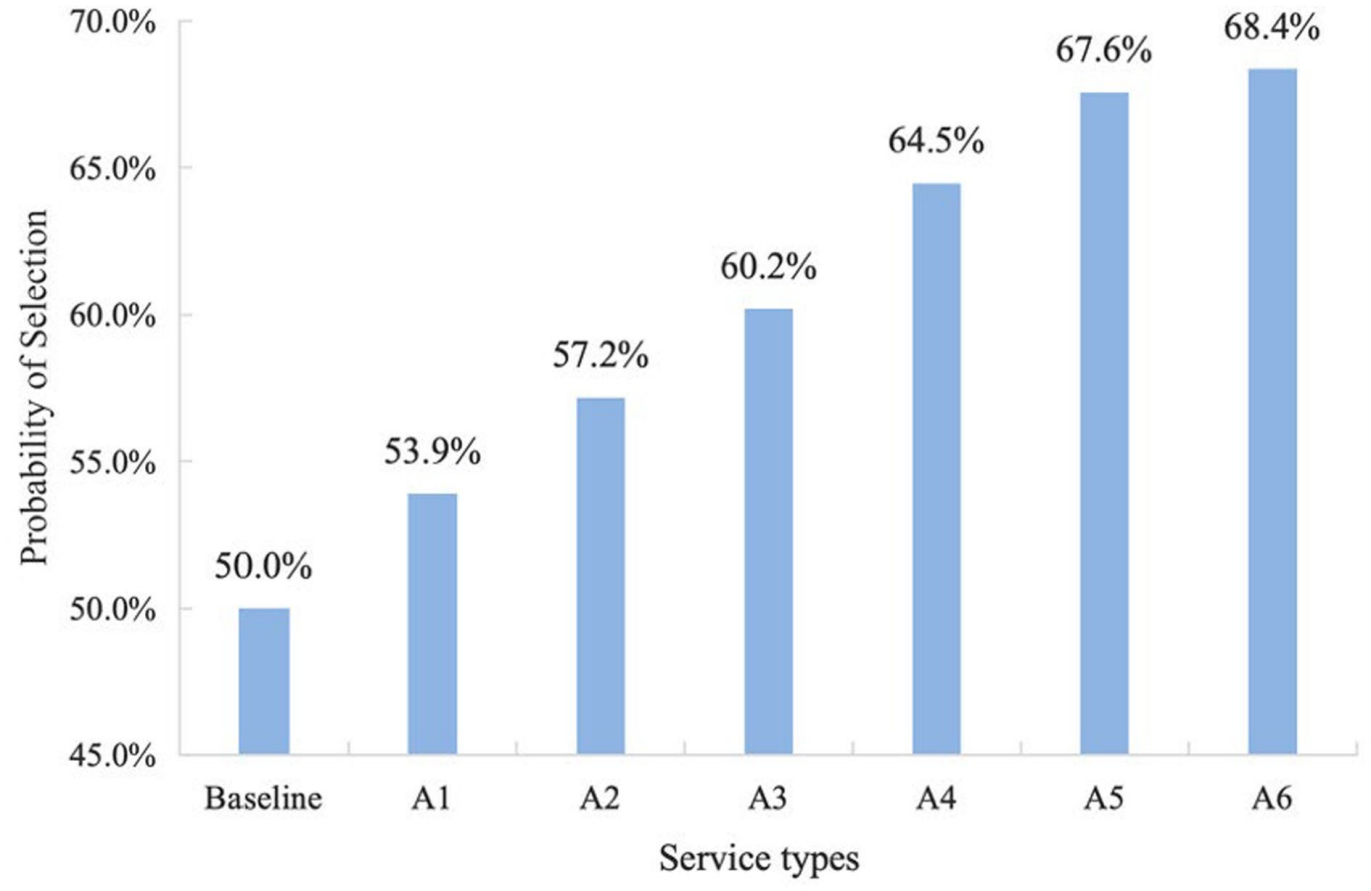
Changes in the probability of IPC selection among patients. Baseline is defined as all other attributes being at the reference level (non-tertiary hospital, attending pharmacist, improved efficacy, 5 min per session, once per week, 10 CNY per session); A1 = Three times per week; A2 = Three times per week + 15 min/session; A3 = Three times per week + 15 min/session + Reduced medication errors; A4 = Three times per week + 15 min/session + Reduced medication errors +Associate Chief Pharmacist; A5 = Three times per week + 15 min/session + Reduced medication errors + Associate Chief Pharmacist + Tertiary Public Hospitals; A6 = Three times per week + 15 min/session + Reduced medication errors + Associate Chief Pharmacist + Tertiary Public Hospitals + 10CNY per session.

Sequentially combining optimal levels across attributes generated the most preferred service configuration (25, 26). Patients were most likely to select consultations delivered by associate chief pharmacists in tertiary hospitals, three times per week, lasting 15 min, and focused on reducing medication errors, with a fee of 10 CNY per session. The predicted choice probability for this optimal combined profile was 68.4% (Figure 4).

### 3.5 Subgroup analyses

Subgroup analyses revealed significant differences in at least one attribute across subgroups defined by residence, household per capita disposable income, and presence of chronic conditions requiring long-term therapy.

#### 3.5.1 Analysis of heterogeneity in preferences based on household *per capita* disposable income

Using the 2024 national per capita disposable income (41,314 CNY) as a benchmark, respondents were stratified into a low-income group (household per capita disposable income <40,000 CNY, *n* = 80) and a high-income group (≥40,000 CNY, *n* = 240). The high-income group placed greater value on hospital tier, demonstrating a significant preference for tertiary public hospitals ( β=0.175 , *p* < 0.001) with a WTP of 6.18 CNY. Conversely, the low-income group showed no statistically significant preference for this attribute ( β=−0.017 , *p* = 0.842), suggesting that this population may prioritize medical accessibility over hospital reputation. Regarding pharmacist professional title, the “Associate Chief Pharmacist” level exerted a significant positive influence on both groups, eliciting high WTP estimates (Low-income: 3.83 CNY; High-income: 5.66 CNY), indicating the universal importance of professional competence across income strata. Distinct divergences were observed in consultation duration and frequency. The high-income group exhibited a strong preference for longer consultations of 15 min/session (WTP = 6.67 CNY), whereas the low-income group showed no significant preference for duration (*p* > 0.05). Regarding frequency, the low-income group favored more frequent visits, showing the highest WTP for five times per week (5.07 CNY). In contrast, the high-income group preferred three times per week (WTP = 4.69 CNY) and displayed no significant preference for the maximum frequency. This suggests that low-income patients may rely on high-frequency contact to safeguard medication safety. Finally, regarding service outcomes, reduced medication errors elicited significant preferences in both groups with narrowed differences in WTP, underscoring medication safety as a core, universally shared priority (Table 6).

**Table 6:** Preference and willingness-to-pay (WTP) for IPC attributes across income-level subgroups.

Attributes and levels	Low-income group (*n* = 80)				High-income group (*n* = 240)			
	*β*	*p*	WTP (CNY)	95% CI	*β*	*p*	WTP (CNY)	95% CI
Hospital tier (Ref: non-tertiary public hospitals)								
Tertiary Public Hospitals	−0.017	0.842	−0.24	−2.07 to 1.59	0.175	<0.001	6.18	3.12 to 9.24
Pharmacist professional title (Ref: Attending Pharmacist)								
Associate Chief Pharmacist	0.265	0.023	3.83	0.63 to 7.03	0.16	0.011	5.66	1.62 to 9.70
Chief Pharmacist	−0.173	0.155	−2.5	−6.00 to 1.00	−0.124	0.055	−4.39	−9.00 to 0.22
Consultation frequency (Ref: once per week)								
Three times per week	0.251	0.03	3.63	0.72 to 6.54	0.133	0.036	4.69	0.72 to 8.66
Five times per week	0.351	0.005	5.07	1.87 to 8.27	0.005	0.945	0.16	−2.80 to 3.12
Consultation duration (Ref: 5 min/session)								
10 min/session	−0.105	0.415	−1.51	−4.80 to 1.78	−0.127	0.054	−4.48	−9.10 to 0.14
15 min/session	−0.007	0.951	−0.1	−2.70 to 2.50	0.189	0.003	6.67	2.83 to 10.51
Service outcome (Ref: improved efficacy)								
Reduced medication errors	0.195	0.017	2.81	0.53 to 5.09	0.097	0.033	3.41	0.45 to 6.37

For the low-income group, the preferred service profile involved associate chief pharmacists providing consultations in non-tertiary hospitals, five times weekly, with 5-min sessions focused on reducing medication errors at 10 CNY per session. The predicted choice probability for this combination was 69.2%. Conversely, high-income patients favored associate chief pharmacists delivering consultations in tertiary hospitals three times weekly, with 15-min sessions prioritizing error reduction at 10 CNY per session. This profile achieved a 67.8% predicted choice probability.

#### 3.5.2 Analysis of heterogeneity in preferences based on residence

Patients were stratified into urban residents (*n* = 250) and rural residents (*n* = 70) based on their permanent residence. Regarding hospital tier, urban residents demonstrated a significant preference for tertiary public hospitals ( β=0.179 , *p* < 0.001), with a WTP of 6.15 CNY. Conversely, rural residents showed no significant preference for this attribute ( β=−0.013 , *p* = 0.882), implying that rural populations may prioritize service accessibility over hospital classification. However, it should be noted that this non-significant result must be interpreted with caution due to the limited sample size of the rural. Regarding pharmacist professional title, urban residents exhibited a significant positive preference for associate chief pharmacist ( β=0.194 , *p* = 0.002; WTP = 6.66 CNY), whereas rural residents showed no significant preference for pharmacist rank (*p* > 0.05; Table 7).

**Table 7:** Preference and willingness-to-pay (WTP) for IPC attributes across residence subgroups.

Attributes and levels	Urban group (*n* = 250)				Rural group (*n* = 70)			
	*β*	*p*	WTP (CNY)	95% CI	*β*	*p*	WTP (CNY)	95% CI
Hospital tier (Ref: non-tertiary public hospitals)								
Tertiary Public Hospitals	0.179	<0.001	6.15	1.39 to 10.91	−0.013	0.882	−0.21	−2.97 to 2.55
Pharmacist professional title (Ref: Attending Pharmacist)								
Associate Chief Pharmacist	0.194	0.002	6.66	0.86 to 12.46	0.169	0.157	2.79	−1.37 to 6.95
Chief Pharmacist	−0.127	0.049	−4.37	−9.44 to 0.71	−0.099	0.413	−1.65	−5.69 to 2.40
Consultation frequency (Ref: once per week)								
Three times per week	0.2	0.001	6.89	0.97 to 12.80	0.032	0.787	0.53	−3.33 to 4.40
Five times per week	0.112	0.089	3.86	−1.16 to 8.88	−0.019	0.880	−0.31	−4.38 to 3.75
Consultation duration (Ref: 5 min/session)								
10 min/session	−0.052	0.432	−1.78	−6.33 to 2.78	−0.409	0.001	−6.77	−12.27 to-1.27
15 min/session	0.221	<0.001	7.61	1.44 to 13.78	−0.182	0.124	−3.02	−7.22 to 1.18
Service outcome (Ref: improved efficacy)								
Reduced medication errors	0.122	0.006	4.19	0.28 to 8.10	0.078	0.358	1.29	−1.55 to 4.12

Residence-stratified analyses revealed that urban residents exhibited a significant negative preference for chief pharmacists ( β=−0.127 , *p* = 0.049; WTP = −4.37 CNY), whereas this preference was non-significant among rural residents (*p* = 0.157). Regarding consultation duration, urban residents showed no distinct preference for a 10-min sessions (*p* = 0.432), while rural residents demonstrated a significant negative preference for this level ( β=−0.409 , *p* = 0.001; WTP = −6.77 CNY).

For the urban group, the most preferred service profile comprised consultations provided by associate chief pharmacists in tertiary hospitals three times weekly, with 15-min sessions focused on error reduction at 10 CNY/session. The predicted choice probability for this combination was 71.4%. Conversely, no statistically significant preferred service profile emerged for rural patients. Given the limited rural subsample (*n* = 70), statistical power may be insufficient to detect subtle preferences. Thus, these non-significant results should not be interpreted as definitive evidence of preference absence or passive acceptance among rural populations.

#### 3.5.3 Analysis of heterogeneity in preferences based on chronic disease status

Patients were stratified into chronic disease (*n* = 100) and non-chronic disease (*n* = 220). The non-chronic group showed no significant preferences for any attribute levels (all *p* > 0.05), except for the 10-min consultation duration. Conversely, chronic disease patients exhibited significant positive preferences for tertiary public hospitals ( β= 0.164, *p* < 0.001; WTP = 4.74 CNY), associate chief pharmacist ( β=0.224 , *p* < 0.001; WTP = 6.49 CNY), a frequency of three times per week ( β=0.157 , *p* = 0.008; WTP = 4.56 CNY), and a duration of 15 min/session ( β=0.150 , *p* = 0.011; WTP = 4.33 CNY). Notably, chronic disease patients demonstrated a significant negative preference for reduced medication errors ( β=−0.116 , *p* = 0.006), indicating that patients with long-standing illnesses prioritized therapeutic efficacy over medication safety (Table 8).

**Table 8:** Preference and willingness-to-pay (WTP) for IPC attributes across chronic disease status subgroups.

Attributes and levels	Chronic disease group (*n* = 100)				Non-chronic disease group (*n* = 220)			
	*β*	*p*	WTP (CNY)	95% CI	*β*	*p*	WTP (CNY)	95% CI
Hospital tier (Ref: non-tertiary public hospitals)								
Tertiary Public Hospitals	0.164	<0.001	4.74	1.45 to 8.03	−0.048	0.664	−0.92	−5.15 to 3.31
Pharmacist professional title (Ref: Attending Pharmacist)								
Associate Chief Pharmacist	0.224	<0.001	6.49	1.94 to 11.05	−0.132	0.396	−2.53	−8.75 to 3.69
Chief Pharmacist	−0.131	0.032	−3.8	−7.71 to 0.11	−0.112	0.474	−2.14	−8.25 to 3.98
Consultation Frequency (Ref: once per week)								
Three times per week	0.157	0.008	4.56	0.56 to 8.55	0.123	0.434	2.35	−3.85 to 8.55
Five times per week	0.053	0.403	1.52	−2.12 to 5.16	0.266	0.093	5.08	−2.21 to 12.38
Consultation Duration (Ref: 5 min/session)								
10 min/session	−0.098	0.114	−2.84	−6.60 to 0.93	−0.372	0.021	−7.11	−15.59 to 1.38
15 min/session	0.15	0.011	4.33	0.42 to 8.24	−0.004	0.979	−0.08	−5.76 to 5.61
Service outcome (Ref: improved efficacy)								
Reduced medication errors	−0.116	0.006	−3.35	−6.22 to −0.48	−0.149	0.177	−2.85	−7.64 to 1.93

Patients with chronic diseases displayed a significant negative preference for chief pharmacist ( β=−0.131 , *p* = 0.032; WTP = −3.80 CNY, 95% CI −7.71 to 0.11), whereas this preference was non-significant among non-chronic patients (*p* = 0.474). Notably, an inverted U-shaped preference emerged for pharmacist seniority: Patients favored associate chief over chief pharmacists, likely due to perceived accessibility and competency sufficiency.

For chronic disease patients, the optimal service profile comprised associate chief pharmacist consultations in tertiary hospitals three times weekly, delivered in 15-min sessions, with an improved-efficacy focus at 10 CNY per session.

## 4 Discussion

In recent years, the Chinese government has consistently promoted the high-quality development of pharmaceutical services. While existing literature has largely focused on service providers (pharmacists) or policy perspectives, empirical research exploring preferences for pharmaceutical services from the patient’s perspective remains scarce. Using a DCE, this study investigated the influence of six key attributes—hospital tier, pharmacist professional title, consultation frequency, duration, service outcome, and cost—on patient preferences for IPC. The findings reveal that patient choices are jointly shaped by economic attributes (cost) and multiple non-economic attributes. Notably, the associate chief pharmacist qualification exerted the most significant positive influence, followed by a frequency of three times/week, tertiary public hospitals, a duration of 15 min/session, and the outcome of reducing medication errors. Conversely, a service cost of 15 CNY/session had a negative impact. These findings align with conclusions summarized in a systematic review by Riboulet et al. (16) Furthermore, this study innovatively clarifies the hierarchy of non-economic attributes and quantifies their relative importance, providing a comprehensive depiction of actual patient preferences under multi-attribute trade-offs. This offers empirical evidence for designing differentiated and precise strategies for IPC.

Regarding pharmacist professional title, respondents demonstrated a positive preference for associate chief pharmacists but, counterintuitively, a negative utility for chief pharmacists. This non-linear trend reveals that patients do not simply equate higher rank with better service; rather, they trade off between professionalism, accessibility, and demand alignment. First, from the perspective of demand-side perception, associate chief pharmacists are often viewed as professionally adequate and more accessible, associated with ample communication time, shorter waiting periods, and more egalitarian interaction. In contrast, chief pharmacists may be inferred as scarce resources associated with higher booking difficulties and time costs. Even though the fee was isolated as a separate attribute in the design, respondents likely linked the chief rank to higher opportunity costs. Second, a distinct over-matching effect exists. In most scenarios, patient needs center on routine medication management and education—tasks of low-to-moderate complexity where a competent is sufficient mindset prevails. Under this cognitive model, associate chief pharmacists are perceived as the appropriate Specialists, whereas chief pharmacists may be viewed as a resource mismatch due to their excessive seniority, triggering a negative psychological reaction. Third, chief pharmacists typically shoulder heavier burdens in medical practice, research, teaching, and administration, leading to immense time pressure that may shorten communication with individual patients. The authoritative persona formed over a long career may result in a direct, concise communication style, which patients might misinterpret as rushed or impatient, whereas associate chief pharmacists—the backbone of the clinical force—may better balance technical expertise with empathetic communication. Additionally, during the pilot phase, some patients struggled to distinguish between ranks (attending, associate chief, chief). Consequently, in the formal survey, we utilised the colloquial term “Specialist” to annotate associate chief pharmacists and “Senior Specialist” (Big Specialist) for chief pharmacists to aid understanding. This labeling might have introduced a value suggestion, creating an impression that an specialist is sufficient, thereby amplifying the negative selection against chief pharmacists. Subgroup analyses showed that while income levels did not sway this preference, the negative preference for chief pharmacists primarily stemmed from urban residents and patients with chronic diseases. These groups, possessing richer medical experiences, have a clearer understanding of their own needs regarding the service outcomes (e.g., technical skills vs. service experience) provided by pharmacists of different ranks, leading to rational trade-offs. Collectively, these results reflect rational patient compromises and suggest that future service implementation should strengthen emotional communication and humanistic care. Methodologically, questionnaire design must rigorously avoid descriptive biases that might interfere with choice behavior.

Patients showed a distinct preference for IPC services provided by associate chief pharmacists in tertiary hospitals, three times per week, lasting 15 min per session, with a primary focus on reducing medication errors, at a price of 10 CNY per session. Contrary to expectations, patients did not universally select the highest level for every attribute; for instance, associate chief pharmacist and three times/week represent intermediate levels. This indicates that patients do not merely pursue the highest tier of service resources but comprehensively weigh service quality, accessibility, and economic burden. Theoretically, this preference pattern can be elucidated by the principle of diminishing marginal utility in medical service demand theory: once service quality reaches a certain threshold, the marginal utility derived from further elevating specifications or frequency diminishes, while the additional time and economic costs may reduce overall satisfaction and compliance. From a practical standpoint, intermediate levels of professional qualification and frequency satisfy core needs (such as reducing medication errors) while avoiding the pressure of time, cost, and resource occupation associated with excessive specifications. This suggests a patient preference for moderate and efficient service models, providing theoretical support for the refined design of future IPC services.

Within the narrowly defined price range of 10 to 15 CNY per session, the out-of-pocket cost exerted a statistically significant yet relatively minor influence on patient choices compared to key non-monetary attributes. Although an incremental price increase decreased the probability of service selection ( β=−0.036 , *p* < 0.001), the absolute magnitude of this impact remained limited. Drawing upon Lancaster’s Characteristics Theory, this dynamic reflects rational attribute trade-offs. Patients do not derive utility merely from the IPC label, but seek to maximize the perceived value of its constituent attributes. They tend to prioritize allocating resources to high-value features—such as tertiary hospitals or optimal visit frequencies—and demonstrate a willingness to pay a premium for these benefits over negligible cost savings. Crucially, however, this localized observation must be strictly contextualized within the current realities of the Chinese healthcare system. For inpatients, a 5 CNY/visit marginal fluctuation represents a negligible fraction of total hospitalization expenses, a perception further mitigated by the expectation of partial reimbursement from the Basic Medical Insurance (BMI) system. Consequently, the limited overall impact of cost observed here is a product of the narrow methodological price bracket and the specific inpatient setting, rather than a generalized patient insensitivity to pricing. Extrapolating these context-dependent findings to broader pricing policies for clinical pharmacy services should therefore be approached with extreme caution, as larger out-of-pocket variations may yield substantially different preference structures.

This study reveals significant heterogeneity in preferences and WTP for IPC across different income levels, demonstrating a clear divergence in the prioritization of service elements. Specifically, the high-income group prioritized hospital tier, consultation duration, and pharmacist professional title, with reduced sensitivity to frequency. This aligns with previous findings that populations with higher socioeconomic status place greater emphasis on the quality and professional level of medical resources (27, 28), further confirming the core role of perceived value in this group’s decision-making. Notably, our findings are consistent with those of Riboulet et al. (16), who identified service duration as a consistent core element influencing pharmacy service preferences, providing direct evidence of the high-income group’s preference for extended consultations and in-depth service experiences. In contrast, the low-income group prioritized service outcomes and frequency, with lower WTP values. This likely stems from a stronger emphasis on economic accessibility and the need to match service frequency to obtain maximized pharmaceutical support within a limited budget. While this supports health economics research suggesting low-income populations are more sensitive to medical service prices (29), our study extends this by revealing that this sensitivity extends beyond WTP into the prioritization of service structural elements, expanding the explanatory boundaries of existing theories.

Preferences also varied significantly by residence. Urban residents demonstrated stable positive preferences for multiple core attributes, whereas rural residents only showed a significant negative preference for 10 min/session, with no significant inclinations for other attributes. The coverage and quality of clinical pharmacy services in China’s county and municipal areas are generally insufficient (30), which may result in limited awareness and experience of such services among rural residents, leading to a lack of clear choice tendencies. This result corroborates the dual gap in both supply and demand for clinical pharmacy services in rural areas, suggesting that policymakers need to strengthen the allocation of pharmacy resources in primary healthcare institutions and enhance patient awareness through health education. Conversely, urban residents showed a significant inclination toward high-level medical institutions, senior pharmacists, longer durations (15 min), and moderate frequency (3 times/week), placing greater importance on reducing medication errors. This suggests that populations with high accessibility to medical resources view pharmacy services as a critical link in ensuring medication safety and optimizing treatment. This likely stems from urban residents’ higher sensitivity to medical safety incidents and a more developed understanding of the pharmacist’s role, providing empirical support for strengthening medication safety management in urban institutions.

Finally, preferences differed by health status. The positive role of pharmacist intervention in chronic disease management is well-established (31, 32). This study found that, compared to patients without chronic conditions, those with chronic diseases exhibited more significant and consistent preferences, particularly for services provided by associate chief pharmacists in tertiary hospitals, three times per week, for 15 min. This contrasts with Wu et al. (28), who reported that chronic patients preferred community hospitals, suggesting that while the community-level treatment capacity for chronic diseases has been recognized under China’s hierarchical diagnosis and treatment system, pharmaceutical service capacity has not kept pace. This discrepancy indicates that during hospitalization, chronic patients exhibit significantly elevated dependence on complex medication management, specialized resources, and multidisciplinary platforms, aligning with existing research on the reliance of chronic patients on multidisciplinary collaboration and high-level resources during inpatient care (32).

Interestingly, patients with chronic diseases showed a significant negative preference for the reducing medication errors attribute. Considering the reference level was improved efficacy, this reflects that these patients perceive enhanced efficacy as having higher value than error reduction. Burdened by long-standing illnesses, the core need of chronic patients is symptom alleviation and quality of life improvement; thus, may exhibit greater risk tolerance to pursue therapeutic gains, valuing effectiveness over safety. In contrast, patients without chronic diseases showed no significant preference here, indicating less rigid demand for IPC. This differentiated demand suggests that IPC implementation should adopt a stratified strategy: providing high-frequency, specialized interventions for chronic patients, while adopting a flexible, on-demand model for non-chronic patients to achieve optimal resource allocation.

Based on these empirical findings, we propose the following policy recommendations. First, optimize resource allocation. In the short term, service frequency and duration should be differentiated based on patient characteristics (disease type, economic level, residence) to guide resources toward attributes with higher clinical value and demand. Long-term strategies should focus on strengthening clinical pharmacy talent pipelines and comprehensive service capabilities in medical institutions to cultivate robust doctor-patient trust. Second, strengthen grassroots pharmacy services. By integrating into national policies on hierarchical diagnosis and chronic disease management, systematic medication education and standardized pharmacy services should be extended to rural settings. This will enhance the awareness and utilization of pharmacy services among rural residents and populations with low health literacy, narrowing the urban–rural gap. Third, incorporate IPC into health insurance reimbursement. Currently, IPC fees are piloted only in select provinces and tertiary hospitals. This study confirms that cost is not the primary barrier to choice. Accordingly, we recommend that healthcare security administrations build upon pilot experiences to progressively expand IPC charging policies to more provinces and healthcare institutions of various levels.

## 5 Limitations

Several methodological and contextual limitations should be acknowledged when interpreting the findings of this study.

Research indicates that pharmacy services significantly reduce drug-related problems in the older population (33), establishing them as a key target population. First, the cognitive demands of DCE questionnaires resulted in underrepresentation of older population and individuals with low educational attainment, which may limit the generalizability of the sample. Compounded by this demographic skew, the limited sample sizes in specific subgroup analyses—particularly the low-income (*n* = 80) and rural (*n* = 70) cohorts—reduced statistical power and widened confidence intervals in the mixed logit models. Therefore, non-significant preferences observed in these subgroups (e.g., apparent indifference to higher-tier hospitals or senior pharmacist titles among rural patients) should be interpreted with caution, as they may reflect type II errors rather than a genuine absence of preference.

Second, these findings should be contextualized within real-world public health conditions. Rural and low-income populations consistently face systemic barriers to accessing high-quality pharmacy services, an inequity that inherently shapes their stated preferences in hypothetical scenarios. Therefore, the lack of statistical significance should not be misconstrued as evidence that these vulnerable populations are merely “passive recipients” of health care.

Third, the service outcome attribute was operationalized as a binary contrast between improved efficacy and reduced medication errors. Although these two goals are closely intertwined in clinical practice, this study framed them as opposing options to meet the structural requirements of the DCE. This represents an artificial trade-off used to elicit stated preferences, which may entail conceptual simplification and should be interpreted cautiously.

Fourth, colloquial annotations for pharmacist professional titles—“Associate Chief Pharmacist (Specialist)” versus “Chief Pharmacist (Senior Specialist)”—may have introduced unintended semantic bias. This labeling may partially explain the unexpected negative preference for chief pharmacists, as respondents might implicitly associate “Senior Specialist” with unmeasured burdens such as poorer accessibility or higher indirect costs.

Future research should address these limitations through larger, strategically stratified cohorts and refined survey instruments to robustly elucidate nuanced preferences across demographic groups.

Despite these limitations, this study provides novel insights by quantifying patient preferences and WTP for IPC from a demand-side perspective, thereby generating empirical evidence for service optimisation. Subgroup analyses further reveal significant preference variations across income levels, residential locations, and health statuses, establishing a scientific foundation for precise, differentiated service strategies.

## Data Availability

The original contributions presented in the study are included in the article/supplementary material, further inquiries can be directed to the corresponding author.
